# The evolution of BDNF is defined by strict purifying selection and prodomain spatial coevolution, but what does it mean for human brain disease?

**DOI:** 10.1038/s41398-022-02021-w

**Published:** 2022-06-22

**Authors:** Alexander G. Lucaci, Michael J. Notaras, Sergei L. Kosakovsky Pond, Dilek Colak

**Affiliations:** 1 Institute for Genomics and Evolutionary Medicine, Science & Education Research Center, Temple University, Philadelphia, PA USA; 2 Center for Neurogenetics, Brain & Mind Research Institute, Weill Medical College, Cornell University, New York, New York, USA; 3 Gale and Ira Drukier Institute for Children’s Health, Weill Cornell Medical College, Cornell University, New York, NY USA

**Keywords:** Genetics, Neuroscience

## Abstract

Brain-Derived Neurotrophic Factor (BDNF) is an essential mediator of brain assembly, development, and maturation. BDNF has been implicated in a variety of brain disorders such as neurodevelopmental disorders (e.g., autism spectrum disorder), neuropsychiatric disorders (e.g., anxiety, depression, PTSD, and schizophrenia), and various neurodegenerative disorders (e.g., Parkinson’s, Alzheimer’s, etc.). To better understand the role of BDNF in disease, we sought to define the evolution of BDNF within *Mammalia*. We conducted sequence alignment and phylogenetic reconstruction of BDNF across a diverse selection of >160 mammalian species spanning ~177 million years of evolution. The selective evolutionary change was examined via several independent computational models of codon evolution including FEL (pervasive diversifying selection), MEME (episodic selection), and BGM (structural coevolution of sites within a single molecule). We report strict purifying selection in the main functional domain of BDNF (NGF domain, essentially comprising the mature BDNF protein). Additionally, we discover six sites in our homologous alignment which are under episodic selection in early regulatory regions (i.e. the prodomain) and 23 pairs of coevolving sites that are distributed across the entirety of BDNF. Coevolving BDNF sites exhibited complex spatial relationships and geometric features including triangular relations, acyclic graph networks, double-linked sites, and triple-linked sites, although the most notable pattern to emerge was that changes in the mature region of BDNF tended to coevolve along with sites in the prodomain. Thus, we propose that the discovery of both local and distal sites of coevolution likely reflects ‘evolutionary fine-tuning’ of BDNF’s underlying regulation and function in mammals. This tracks with the observation that BDNF’s mature domain (which encodes mature BDNF protein) is largely conserved, while the prodomain (which is linked to regulation and its own unique functionality) exhibits more pervasive and diversifying evolutionary selection. That said, the fact that negative purifying selection also occurs in BDNF’s prodomain also highlights that this region also contains critical sites of sensitivity which also partially explains its disease relevance (via Val66Met and other prodomain variants). Taken together, these computational evolutionary analyses provide important context as to the origins and sensitivity of genetic changes within BDNF that may help to deconvolute the role of BDNF polymorphisms in human brain disorders.

## Introduction

Brain-derived neurotrophic factor (BDNF) is one of the most ubiquitously studied molecules in modern neuroscience [1]. BDNF is a neurotrophin that binds with high affinity to its cognate tyrosine kinase receptor, TrkB [2], to elicit rapid induction of synaptic plasticity [3–5] and neuronal spine remodeling [6, 7]. Additionally, BDNF has been implicated in a variety of brain disorders [1], including depression [8–10], PTSD [11–14], schizophrenia [9, 15–17], Parkinson’s disease [18, 19], and autism spectrum disorders [20–22] amongst many more. BDNF has correspondingly been the primary target, or an ancillary factor, of many novel therapeutics including small molecule mimetics [23, 24] and existing drugs (e.g., antidepressants [25, 26]). Yet, nascent research has provided the humbling reminder that much remains to be discovered about BDNF. In recent years, new BDNF ligands have been discovered [27], new receptor interactions unveiled [27, 28], and mechanisms of behavioral function unlocked [7]. This is a timely reminder that while BDNF has remained a seminal molecule of interest across the broader neuroscience literature, much remains to be discovered about its origins, evolution, function, and disease relevance.

### A primer of the molecular biology of BDNF and its functional topology

BDNF is encoded by the *BDNF* gene [29], whose expression is regulated in humans by an antisense gene (*BDNF-AS*) that can form RNA-duplexes to attenuate translation [30]. Thus, the natural antisense for BDNF is capable of directly downregulating endogenous expression on demand [31]. The *BDNF* gene in humans comprises 11 exons [30] and can produce at least 17 detectable transcript isoforms [29]. Different transcripts are induced in response to activity and/or cellular states, allowing the *BDNF* gene to adjust to environmental stimuli and potential selection pressures. However, all transcripts ultimately yield a singular preproBDNF protein that (prior to intracellular processing, cleavage, and transport) can be partitioned into three domains [11, 29]: a signal peptide, a prodomain, and the mature domain. The signal domain is only 18 amino acid residues long (with ambiguously defined functionality) with the majority of BDNFs functional outputs reflecting sequence specificity to the prodomain and mature domain. The BDNF prodomain encodes binding sites for intracellular transport of both *BDNF* mRNA [32] and BDNF protein [33], and contains numerous posttranslational modification sites [29]. The BDNF prodomain is also the resident location of a widely studied Single Nucleotide Polymorphism (SNP) in neuroscience (Val66Met, or rs6265) [1], and the Furin consensus sequence (Arg 125) for cleavage to its mature form (including by plasmin [34]). The prodomain is composed of 110 amino acids within the N-terminus, and must be processed via proteases to generate mature BDNF [5]. The mature domain of BDNF is almost exclusively composed of the Nerve growth factor (NGF) domain and is responsible for the canonical trophic actions associated with BDNF (e.g., long-term potentiation, rapid-acting antidepressant effects, etc.). Following intracellular handling, processing, and transport, the preproBDNF isoform is cleaved to yield the mature BDNF peptide (which only contains the mature NGF domain). For many years the prodomain was thought to be degraded following the facilitation of BDNF trafficking. However, recent work has shown that the cleaved prodomain can be secreted and bind as a ligand to novel receptors (e.g., SorCS2) [27]. Thus, the BDNF prodomain can influence brain circuits as well as behavior [7]. For a comprehensive, detailed, analysis of the various intricacies of the BDNF gene, protein, and its regulation, more information is provided in [29].

### The conservation of BDNF and neurotrophins: a signal that evolution is important

One of the interesting curiosities surrounding BDNF is its relationship to other neurotrophic (NT) growth factors, comprising NGF, NT-3, and NT-4. Specifically, all neurotrophins retain some intercalated functionality. Neurotrophins also share some commonalities in structure (pre-, pro-, and mature-domains) [29], posttranslational modification potential (e.g., glycosylation [35]), as well as catalytic processing, trafficking, and composition [36]. Specifically, neurotrophins share approximately 50% sequence homology [29], and a comparison of domains and motifs reveals that each comprises a prototypic NGF domain as the principal component of the mature pro-growth peptide (see PFAM database [37]). While each neurotrophin elicits functionality via binding to cognate receptors, neurotrophins also exhibit cross-affinity amongst neurotrophin receptors [38] presumably due to their high rates of structural homology. Not surprisingly then, there is some redundancy in the trophic effects of neurotrophins, yet each still maintains nuanced functionality which remains specific to each factor during central nervous system development [39]. Differences in the evolution and temporal dynamics of regulatory sequences, which target gene products to specific destinations within cell-compartments (e.g., dendrites) [40] or to processing routes (e.g., the activity-dependent release pathway) which alter secretory dynamics and/or bioavailability [41], likely contribute to both similarities and differences between neurotrophins. However, almost nothing is known about how the BDNF prodomain has evolutionarily adapted to specifically regulate BDNF dynamics. While evolution has almost certainly shaped the sequences, structure, and function of BDNF, the modeling of such remains relatively unexplored but could provide important insight into the phylogenetic evolutionary history of BDNF, its selection pressure sensitivity across lineages, and quantitative metrics of evolutionary change across species.

### Purpose of this Study

Here, we use computational methods to explore the molecular evolution of BDNF. To reconstruct phylogenetic trees of BDNF, we utilized sequence alignments of over 160 mammalian species (all available mammalian sequences) to determine the genomic attributes of BDNF evolution that are specific to *Mammalia*. This analysis was specific by being constrained to sequences that have the most direct evolutionary relevance to humans. Notably, we sought to identify sites in BDNF that are subject to pervasive (i.e., consistently across the entire phylogeny) diversifying selection (FEL) or pervasive/episodic (i.e., only on a single lineage or subset of lineages, diversifying selection (MEME). Likewise, utilizing multiple models for the inference of selective pressure and the evaluation of evolutionary change, we identify novel sites within the BDNF prodomain and mature peptide coding regions that are susceptible to synonymous and nonsynonymous changes. Additionally, we investigate which sites in BDNF may be coevolving (BGM). Taken together, these computational evolutionary analyses provide an important context as to the origins and sensitivity of genetic changes within the BDNF gene, which may be important for providing insight into genetic risk factors linked to disease in humans.

## Results

We find that unique evolutionary pressures have shaped the BDNF gene across time. These forces have mostly operated through strict purifying selection. Of note, BDNF elicits tight regulation and specific functionality that can be separated from other neurotrophins, yet these growth factors remain closely related in their structure and sequence, especially in the conserved NGF domain.

### Evolutionary history of mammalian BDNF

Prior to conducting our primary evolutionary analysis, we ported our mammalian species into a platform (*timetree.org*, see refs. [42, 43]) to examine the epoch events that may have influenced the analysis described here. This was an important pre-analysis step to frame the age of our genomes, and the broad-stroke evolutionary pressures that these species have been exposed to (which, in theory, could contribute to subsequent purifying selection and coevolution analyses). As expected, this revealed BDNF as an ancient gene that has been preserved throughout the mammalian lineage and has both survived and been shaped under all major evolutionary events of the past ~177 million years (data not shown). We identified several examples of species-level evolutionary epochs that cross-referenced with major earth events (e.g., bottleneck events) that have historically been believed to drive evolutionary adaptation. This included major geologic periods that are cross-referenced against earth impacts, oxygenation changes across time, atmospheric carbon dioxide concentrations, and solar luminosity. This indicates that even under extreme evolutionary pressures, the BDNF gene has exhibited (relatively speaking) very specific adaptation events (see results below) over millions of years within *Mammalia*. This tracks with the idea that “old genes” tend to be highly conserved, evolve more slowly, and therefore are more likely to exhibit both specific and selective changes as opposed to more dramatic permutations (e.g., gene duplications, etc.).

### Predominant purifying selection in BDNF

A common approach to gain an increased understanding of the evolutionary forces that have shaped proteins is to measure the omega ratio ω consisting of the nonsynonymous (β or dN) and synonymous (α or dS) substitution rates, with ω = β/α for each site in a particular gene of interest [44]. We define two major changes for the amino acid being coded for at each site: synonymous changes, which keep the same amino acid coded for at a particular site, and nonsynonymous changes, which change the amino acid coded for at a particular site. Non-synonymous changes can have strong influences on the structural, functional, and fitness measures of an organism. This is in contrast to synonymous changes which leave the amino acid at a particular site unchanged but can confer weak fitness effects through the emergent properties of codon usage bias, mRNA structural stability, translation, and tRNA availability. However, synonymous changes are typically understood to represent neutral selection acting on coding sequences and provide a baseline rate against which nonsynonymous evolutionary rates can be compared. The omega ratio ω of relative rates of nonsynonymous and synonymous substitutions is a common measure in evolutionary biology of the selective pressure acting on protein-coding sequences. These estimates provide increased information availability as to the type of selection (positive, with omega >1 or negative, with omega <1, or neutral with omega =1) that has acted upon any given set of protein-coding sequences.

**Fig. 1:**
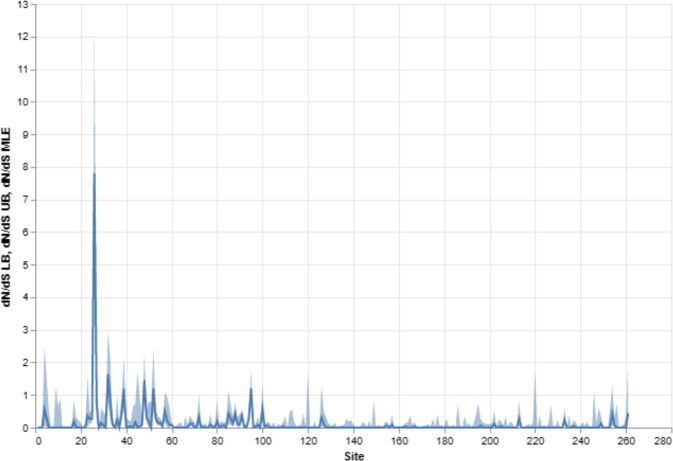
Mammalian BDNF exhibits overwhelmingly strict purifying selection, but also evidence of specific evolutionary pressures at particular sites. The FEL analysis of the BDNF gene found 174 of 261 (66.7%) sites to be statistically significant (LRT *p* value ≤ 0.1) for pervasive negative (purifying) selection. We plot the estimated values of omega (dN/dS) for each site in the alignment. Additionally, we plot 95% confidence intervals (CI) for each site. These results are also available in Table S1. We observe a high degree of strict purifying selection in the Human NGF region. The region for Human NGF corresponds to alignment sites 144–254 (NP_001700.2 and https://github.com/aglucaci/AnalysisOfOrthologousCollections/blob/main/tables/BDNF/BDNF_AlignmentMap.csv). This alignment of BDNF across all selected species (*Mammalia*, see Table 3) reveals a site-specific positive/adaptive diversifying selection and negative purifying selection. The thick line represents the point estimate (i.e., the evolutionary pressure) and the shadings reflect 95% confidence intervals which relate to the upper and lower bound of the point estimates. As shown, the prodomain sites exhibit more pervasive/episodic and positive/diversifying evolutionary selection, consistent with the fact that more disease-associated SNPs occur in this topological region of the BDNF gene in humans (early prodomain mapping not further shown due to nuanced variation across mammalian species).

As FEL analysis is a sensitive measure of negative (purifying) selection, for this analysis we observe a predominant amount of purifying selection (over 66% of sites, 174 sites out of 261; Table S1) in our recombination-free alignment for BDNF. The dN/dS estimates for the entire alignment were plotted including 95% lower- and upper-bound estimates (see Fig. 1 or Table S1). Overwhelmingly, the mature NGF domain of the BDNF exhibited evidence of greater pervasive negative purifying selection relative to the prodomain region of BDNF. Thus over the evolutionary history of *Mammalia*, negative selection has predominantly occurred in the regions of BDNF that encode the functional mature protein that binds TrkB to elicit neurotrophic effects. The mature domain of BDNF has exhibited remarkable conservation across innumerous epochs that have been defined by rapid evolutionary adaptation in other genes and taxa.

### Specific sites that are evolving non-neutrally

To examine specific sites for episodic adaptive evolutionary selection, we utilized an algorithm known as MEME which is fundamentally similar to our FEL analysis (described above) except that it applies a more sensitive method for the detection of both pervasive (persistent) and episodic selection (transient selection occurring only on one or a subset of branches in the phylogenetic tree) as compared to only pervasive selection which occurs across all branches of the phylogenetic tree. Essentially, only a subset of the lineages (i.e., species) are affected allowing for a more granular/sensitive method of detecting selection (whereas FEL is better geared towards broad changes). This analysis revealed that for all sites, only 2.3% (6 of 261; see Table 1) exhibit evidence for episodic diversifying selection (i.e., positive selection) in at least one branch within the phylogeny. Spatially, these mutations occur outside of the NGF functional region of BDNF. Further, this result is essentially relevant as the MEME analysis is a sensitive measure of episodic selection. The sites we observe as statistically significant were 26, 27, 30, 38, 249, and 254. For comparison, these specific sites were realigned to the respective human sites with indel (insertion/deletion) events accounting for any respective discrepancy in specific site numbers. When mapping these sites to the human BDNF coordinate system, they correspond to sites 26, 27, 29, 36, 238, and 240, respectively.

**Table 1:** MEME analysis of the BDNF gene found 6 of 261 (2.3%) of sites to be statistically significant (LRT *p* value ≤ 0.1).

#	CodonSite	Human CodonSite	alpha	beta-	*p*-	beta+	*p*+	LRT	*p* value	# branches under selection	MEME LogL	FEL LogL	Omega
1	26	26	0.14	0.00	0.02	1.12	0.98	6.89	0.01	9	−86.53	−86.53	7.9
2	27	27	0.25	0.08	0.99	28.91	0.01	4.30	0.05	1	−39.25	−37.01	117.6
3	30	29	0.00	0.00	0.00	0.38	1.00	4.63	0.05	6	−35.88	−35.88	inf
4	38	36	0.72	0.00	0.93	8.07	0.07	3.80	0.07	5	−69.22	−66.28	11.3
5	249	238	0.92	0.00	0.99	203.11	0.01	8.25	0.01	1	−50.74	−44.13	221.6
6	254	240	0.23	0.00	0.99	1357.12	0.01	21.81	0.00	1	−44.50	−31.95	5850.4

### Evidence of coevolutionary forces

**Fig. 2:**
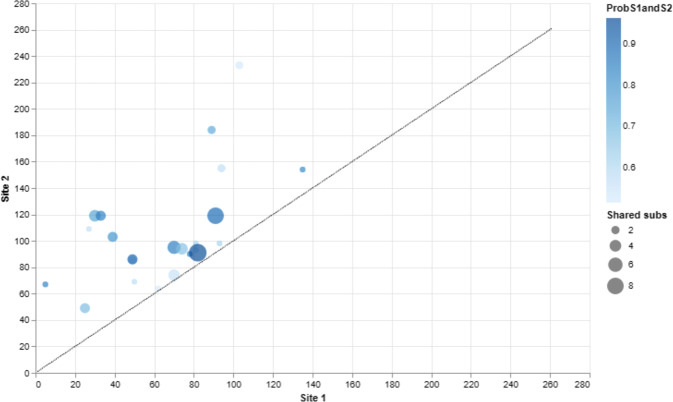
The BGM analysis of BDNF found 23 pairs of coevolving sites out of 261 total sites to be statistically significant (with a posterior probability threshold of 0.5). Here, we plot only the statistically significant coevolving pairs. The number of shared substitutions between pairs of coevolving sites is visualized by the size of the circle, with larger circles indicating more shared substitutions. Poster probability of the interaction (coevolving pair) corresponds to the color blue, with dark blue indicating higher values. Individual BDNF sites are mapped on both the X and Y axis so that readers can view which sites are coevolving with another. Once more, note that the coevolution tends to be focally constrained to the broader BDNF prodomain region at a topological level, which is once more consistent with the idea that the NGF domain (site >144; see Fig. 3) is highly conserved and probably deleteriously impacted by variation. However, we did discover four sites of coevolution in the NGF domain (basically, the mature BDNF protein) that are evolving with early prodomain sites. This highlights that both proximal and distal sites in BDNF can, and indeed are, evolving together over time.

**Fig. 3:**
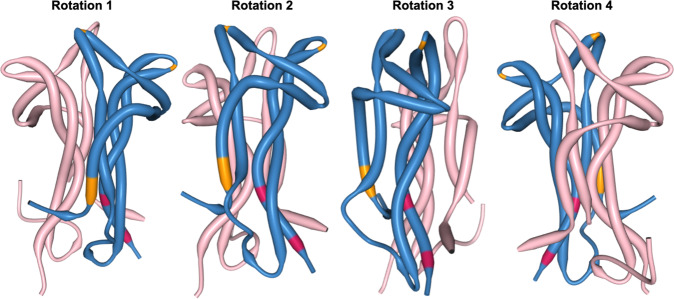
BDNF NT-4 heterodimer structural analysis to highlight coevolving and adaptive sites at the 3D protein level. Demonstrating the structural configuration of the BDNF (blue) and NT-4 (pink) heterodimer (see https://www.rcsb.org/structure/1B8M), with rotations (arbitrary degrees) shown to accentuate the view of coevolving sites (orange, see also Fig. 2 and Table 2) and positively evolving sites (red; see Table 1). The PDB structure is limited to the NGF domain which limits our ability to highlight sites of interest (SOI), therefore we have limited our annotation only to the modeled sites in the structure. The relative positioning of coevolving and positively evolving sites in this heterodimer visualization are in proximity to looping and other macro tertiary structures of protein. An interactive figure that is rotatable in 3D space, where occupations occur in three dimensions (i.e., teasing out relative proximity in 2D linear space), is available here: https://observablehq.com/@aglucaci/bdnf-structure.

To examine the coevolution of sites, i.e., if one particular amino acid was evolving in-tandem with another, we subjected our protein-coding gene sequences to the BGM algorithm which leverages Bayesian graphical models [45]. The BGM algorithm infers substitution history through the use of maximum-likelihood analyses for ancestral sequences and maps these to the phylogenetic tree, which allows for the detection of correlated patterns of substitution [45]. For our BGM analysis, we find evidence for 23 pairs of coevolving sites. This suggests interaction dynamics in tertiary space of the 3D, folded, protein level (see relevant sites in Fig. 2) BDNF protein structure. Alternatively, this data may be evidence that coevolving sites may be related to other fitness consequences (e.g., compensation) for maladaptive changes in another part of the protein sequence that may have occurred. When we review these sites, we notice that several pairs (see Fig. 2) occur within alignment sites, which correspond to the Human BDNF coordinate system (Table 1). These include pair-sites of (89, 184), (94, 155), (103, 233), and (135, 154). Of note, several other sites also display interesting geometric features including triangular relations [(81, 93), (93, 98), and (81, 98)], an acyclic graph network of site connections [(70, 74), (74, 94), (94, 155) and (25, 49), (49, 85), (49, 86)], more complex double-linked coevolutionary sites [(39, 103) and (103, 233)], and triple-linked coevolutionary sites [(30, 119), (33, 119), and (91, 119)]. Additionally, three-dimensional reconstruction—here focusing on a specific heterodimer configuration of BDNF and NT-4 as an example of a spatial protein–protein interaction—highlights that coevolving sites, as well as positively evolving sites, are likely to have been fine-tuned over time to help support BDNF’s cognate functionality (see Fig. 3). Mapping our FEL purifying sites in a structural configuration was not shown due to the overwhelming nature of negative selection acting on BDNF within mammals.

## Discussion

In this study, we explore the evolutionary history of the BDNF gene in *Mammalia*. The BDNF gene is implicated in a number of human diseases including a variety of brain disorders such as neurodevelopmental disorders (e.g., autism spectrum disorder), neuropsychiatric disorders (e.g., depression, PTSD, and schizophrenia), and some neurodegenerative disorders [1]. By using orthologous BDNF sequences within the *Mammalia* taxonomic group, our results indicate that unique site-specific changes within BDNF have evolved over time. We performed a number of comparative evolutionary analyses to tease out signals from our orthologous gene collection in BDNF. Of note, the BDNF gene elicits tight regulation and specific functionality that can be separated from other neurotrophins, yet these growth factors remain closely related in their structure and sequence and conservation of the NGF functional domain. In the NGF domain, we observe a high degree of conservation (via purifying selection) across species, owing to the functional importance of this region in protein–protein interactions. This work additionally provides broad comparative insights into the evolutionary history of the BDNF gene family. Our MEME method identified novel substitutions (see Table 1) in regions of BDNF that may provide significant areas of interest for designing molecular therapeutic approaches, and their potential broader significance are outlined in further detail below.

### Predominant purifying selection across BDNF in Mammalia

Over time, evolution drives the divergence of genetic sequences, but what can we learn from the direct comparison of the sequences of the BDNF gene in *Mammalia*? By comparing the BDNF products of orthologous sequences in different species, we observe the accumulation of mutations at different sites with varying degrees of insight into both BDNF functionality (see [29] for site annotation) and potential disease [1]. These are summarized in full within Table S1 and Table 1. Coding sequences with highly constrained structures are expected to fix nonsynonymous mutations at a slower rate due to the maladaptive nature of changes such as what we observe with FEL negatively selected sites across BDNF. Additionally, we observe a high degree of negative (purifying) selection across the main functional domain (NGF) of BDNF. While structures for the NGF domain in most species under analysis do not exist, based on our findings we expect a highly conserved tertiary structure. Based on the high degree of purifying selection observed across BDNF, we hypothesize that BDNF plays a critical role in the underlying network of genes governing homeostasis and normal organismal development. This may have happened because BDNF is particularly useful specifically for the phylogenetic branch in question (i.e., mammals). This interpretation is also consistent with the observation that BDNF is essential for normative development and is lethal in non-conditional full knock-out mammalian models.

### Non-neutral positive diversifying evolution sites in the BDNF gene

It has been described that BDNF plays a particular role as a foundational gene for brain development [11]. Despite a significant level of purifying selection shaping the evolutionary history of BDNF (Fig. 1), we observe several novel statistically significant sites under positive episodic diversifying selection across the BDNF gene (see Table 1). Traditionally, the evolution of this variety consists of amino acid diversifying events that may promote phylogenetic adaptation and/or functionality. These results are entirely novel—they have not been previously reported (to the best of our knowledge) and MEME is an established and sensitive method for the analysis of episodic diversifying sites. Thus, the very specific and limited sites within BDNF to exhibit such patterns is a highly promising result from which to further disentangle BDNF's complex functionality and disease linkage. We would encourage biologists to consider these sites as those that may contain important adaptive functions within the BDNF gene. However, where our results fall within the context of a core protein–protein interaction network of required genes for neural cellular diversity and development is yet to be determined. We do note that at least one identified site (238) overlaps with potential posttranslational modifications to the human BDNF peptide (specifically, a disulfide bridge; see UniProt and [29]). This supports the idea that non-neutral positive diversifying sites within BDNF are not spurious and likely reflect specialized, regulatory, or functional capacities that may have yet to be annotated in full. Given that this manuscript is devoted to the analysis of BDNF’s evolution in mammals, we highlight the potential importance of these sites but emphasize that their importance remains a hypothesis that should be tested in well-defined experiments under controlled laboratory conditions.

### Discovery of proximal and distal coevolving site-pairs in the BDNF gene

Another novel, and potentially important, series of findings in this manuscript was the presence of numerous sites that exhibit coevolution. In fact, we observe a significant number of coevolving sites within the BDNF gene (see Fig. 2 and Table 2), and these too reflect an entirely novel aspect of BDNF biology that has not previously been reported. Evidence of coevolving sites are not limited to a particular domain (e.g., prodomain vs mature) nor specific motifs. Instead, coevolving pairs seem to be distributed across the entirety of BDNF with, perhaps unsurprisingly, an increased density of interactions early in the prodomain region. However, we also note that there are coevolving sites in the mature NGF domain which are “linked” to early domain sites. Importantly, these relationships may confer strong epistatic interactions shaping the continued evolution of this critically important gene. The new evidence for coevolution may point to the importance of these sites in shaping the early regulatory or main functional (NGF) domain of BDNF. These residues may form important interactions for the functional integrity of BDNF and, importantly, the highly specific pairs which span the BDNF prodomain and its mature region point to a new mechanism by which the BDNF prodomain may have coregulated the mature domain (or vice versa). Alternatively, these coevolving pairs may be part of a network of residues occupying a shifted fitness landscape in order to accommodate new or species-specific functional requirements.

**Table 2:** The BGM analysis of BDNF found 23 pairs of coevolving sites out of 261 total sites to be statistically significant (with a posterior probability threshold of 0.5).

#	Site 1	Site 2	P [Site 1 –> Site 2]	P [Site 2 –> Site 1]	P [Site 1 < –> Site 2]	Site 1 subs	Site 2 subs	Shared subs
1	5	67	0.52	0.32	0.84	1	1	1
2	25	49	0.022	0.67	0.69	8	6	3
3	27	109	0.12	0.45	0.57	2	1	1
4	30	119	0.2	0.56	0.75	6	12	4
5	33	119	0.12	0.72	0.84	3	12	3
6	39	103	0.22	0.58	0.8	8	4	3
7	49	85	0.48	0.049	0.53	6	3	2
8	49	86	0.36	0.55	0.91	6	5	3
9	50	69	0.31	0.27	0.58	1	2	1
10	62	64	0.25	0.27	0.52	1	1	1
11	70	74	0.17	0.38	0.55	7	14	4
12	70	95	0.59	0.26	0.85	7	21	5
13	74	94	0.034	0.64	0.68	14	7	4
14	78	90	0.46	0.38	0.84	1	1	1
15	81	93	0.27	0.31	0.58	1	1	1
16	81	98	0.27	0.33	0.61	1	1	1
17	82	91	0.045	0.92	0.96	15	29	9
18	89	184	0.24	0.5	0.74	2	6	2
19	91	119	0.22	0.67	0.89	29	12	8
20	93	98	0.31	0.3	0.61	1	1	1
21	94	155	0.28	0.29	0.57	7	2	2
22	103	233	0.4	0.13	0.53	4	3	2
23	135	154	0.4	0.43	0.82	1	1	1

### Potential structural implications of evolving sites

In considering our observation of both diversifying selection and coevolving sites in the BDNF gene, we considered the potential implications this may have at a protein structural level in three-dimensional space (see Fig. 3). While protein structural impacts from evolution remain poorly understood and cannot be completely experimentally disentangled in a confirmatory sense, the implications fall upon our understanding of basic BDNF neurobiology. Here we note that our BGM and FEL analyses implicate the prodomain—the primary topological region of BDNF known for polymorphic variability (e.g., Val66Met and Gln75His) that is often linked to disease [1, 11, 29], and our 3D modeling suggests that two of our coevolving sites appear to be associated with looping structures that could have important yet to be discovered functionality. In this regard, we predict that the evolutionary changes described here are likely to reflect some form of specialization and/or divergence in function and/or interaction partners at different points of BDNF’s evolutionary history in mammals. Thus, further work may unveil yet more novel sites that could provide further insight into the origins of BDNF’s diverse functionality and its role in disease.

### Limitations of our computational evolutionary analysis

This analysis focused on BDNF sequences contained in the taxonomic group *Mammalia* in lieu of examining a more inclusive dataset for BDNF containing sequences from all of *Gnathostomata* (jawed vertebrates) or extension into invertebrate clades which may contain BDNF or BDNF-like analog genes. Our results are applicable to mammals, which are our intentional taxonomic group of study, but we nonetheless emphasize that our results do not capture the *entirety* of BDNF’s evolutionary history (e.g., there could be more to learn about BDNF from birds, lizards, fish, and higher-order taxonomic groups which we do not evaluate here). In addition, we do not explore the patterns or mutational processes occurring outside of coding-sequence evolution which include complex structure and dynamics of non-coding regions in the BDNF gene. Therefore, evolutionary temporality is important in the context and interpretation of our results because *Mammalia* represents only a portion of the long evolutionary history of BDNF. Although we failed to find evidence for recombination in our dataset, species where we may find evidence for recombination may have been precluded from our analysis due to our decision to focus on mammalian BDNF evolution. Further, a limitation of the current analysis is owed to the presence of indel events, especially in the early region of the alignment but which also occur in other spatially distributed regions of the BDNF gene. These indel events are not currently modeled in existing codon substitution models but may represent an additional pathway of evolutionary change. Nonetheless, the prominence of indels in our observations indicates that several regions of BDNF may evolve significantly through indel events across species. Lastly, although there is a risk that the “gappy” nature of the early region of our multiple sequence alignment may be a computational artifact of the alignment procedure, based on all other outputs we believe that our results are reasonably interpreted and have subsequently tolerated these potential effects.

### Future directions: understanding the remainder of the neurotrophin family

We hypothesize that the similarities between neurotrophins reflects conserved evolutionary selection for motifs and domains which support common functionality in neurotrophic factors between sites and lineages. While we note significant isotropy in mature peptide sequences for these factors, anisotropic pressures likely influenced the prodomain sequences of neurotrophins leading to alterations in processing, trafficking, regulation, and secretion. As such, we also predict differences in the evolutionary fate of other neurotrophins which also exhibit compartmentalized functionality due to similar alterations within their prodomains (i.e., similar results may be reasonably anticipated in NGF, NT-3, and NT-4).

## Conclusion

To sum up, our research modeled the natural evolutionary history of changes in BDNF across >160 mammalian genomes. Conservatively, this analysis spans ~177 million years of evolution—and going deeper could yet reveal more information on the ontogenesis of BDNF and its topological structure (and, consequently, function). Notably, we observed strict purifying selection in the main functional domain of the BDNF gene in mammals and discovered 6 specific sites in our homologous alignment that are under episodic selection in the early regulatory region of BDNF (i.e., the prodomain) and in the terminal region of BDNF. We also make the case for spatial coevolution within this gene, with 23 pair-sites that have evolved together. In sum, these data go above and beyond the common trope that “BDNF is highly conserved” by defining exactly where and how the mammalian BDNF has evolved. Thus, we confirm the widespread belief that the BDNF prodomain is more prone to change than the mature BDNF protein, having important implications for how we think about and consider genetic variation in BDNF and its linkage to disease.

## Methods

### Data retrieval

For this study, we queried the NCBI Ortholog database via https://www.ncbi.nlm.nih.gov/kis/ortholog/627/?scope=7776. For the purpose of this study, as we are interested in mammalian BDNF evolution, we limited our search to only include species from this taxonomic group (mammals, *Mammalia*). This returned 162 full gene transcripts and protein sequences. We downloaded all available files: RefSeq protein sequences, RefSeq transcript sequences, Tabular data (CSV, metadata). In Table 3, we provide a table of the species included in this analysis but we also make this accessible via GitHub. Furthermore, we also make these species NCBI accessions (see also Table 3) available for download on GitHub:

- AnalysisOfOrthologousCollections/BDNF_orthologs.csv at main · aglucaci/AnalysisOfOrthologousCollections · GitHub

**Table 3:** Tabulation of species included in our analysis, comprising NCBI ortholog gene IDs, symbols, mammalian species, common name, and RefSeq accessions.

Gene ID	Gene symbol	Description	Scientific name	Common name	RefSeq transcript accessions	RefSeq protein accessions
627	BDNF	brain-derived neurotrophic factor	*Homo sapiens*	human	NM_001709.5	NP_001700.2
12064	Bdnf	brain-derived neurotrophic factor	*Mus musculus*	house mouse	NM_001048142.1	NP_001041607.1
24225	Bdnf	brain-derived neurotrophic factor	*Rattus norvegicus*	Norway rat	NM_001270630.1	NP_001257559.1
397495	BDNF	brain-derived neurotrophic factor	*Sus scrofa*	pig	XM_005654684.3	XP_005654741.1
403461	BDNF	brain-derived neurotrophic factor	*Canis lupus familiaris*	dog	XM_038429434.1	XP_038285362.1
493690	BDNF	brain-derived neurotrophic factor	*Felis catus*	domestic cat	NM_001009828.1	NP_001009828.1
503511	BDNF	brain-derived neurotrophic factor	*Pan troglodytes*	chimpanzee	NM_001012441.1	NP_001012443.1
554233	BDNF	brain-derived neurotrophic factor	*Monodelphis domestica*	gray short-tailed opossum	XM_007497196.2	XP_007497258.1
617701	BDNF	brain-derived neurotrophic factor	*Bos taurus*	cattle	XM_005216334.4	XP_005216391.1
701245	BDNF	brain-derived neurotrophic factor	*Macaca mulatta*	Rhesus monkey	XM_015114598.2	XP_014970084.1
100009689	BDNF	brain-derived neurotrophic factor	*Equus caballus*	horse	NM_001081787.1	NP_001075256.1
100081142	BDNF	brain-derived neurotrophic factor	*Ornithorhynchus anatinus*	platypus	XM_029059317.2	XP_028915150.1
100356949	BDNF	brain-derived neurotrophic factor	*Oryctolagus cuniculus*	rabbit	XM_017345633.1	XP_017201122.1
100409412	BDNF	brain-derived neurotrophic factor	*Callithrix jacchus*	white-tufted-ear marmoset	XM_009007854.3	XP_009006102.1
100447350	BDNF	brain-derived neurotrophic factor	*Pongo abelii*	Sumatran orangutan	XM_002821931.2	XP_002821977.1
100467162	BDNF	brain-derived neurotrophic factor	*Ailuropoda melanoleuca*	giant panda	XM_011226480.3	XP_011224782.2
100594402	BDNF	brain-derived neurotrophic factor	*Nomascus leucogenys*	northern white-cheeked gibbon	XM_003254347.2	XP_003254395.1
100667885	BDNF	brain-derived neurotrophic factor	*Loxodonta africana*	African savanna elephant	XM_023550772.1	XP_023406540.1
100730257	Bdnf	brain-derived neurotrophic factor	*Cavia porcellus*	domestic guinea pig	XM_013147262.2	XP_013002716.1
100768664	Bdnf	brain-derived neurotrophic factor	*Cricetulus griseus*	Chinese hamster	XM_007653166.4	XP_007651356.1
100934810	BDNF	brain-derived neurotrophic factor	*Sarcophilus harrisii*	Tasmanian devil	XM_031944159.1	XP_031800019.1
100958946	BDNF	brain-derived neurotrophic factor	*Otolemur garnettii*	small-eared galago	XM_012814047.1	XP_012669501.1
100983866	BDNF	brain-derived neurotrophic factor	*Pan paniscus*	pygmy chimpanzee	XM_034932318.1	XP_034788209.1
101007866	BDNF	brain-derived neurotrophic factor	*Papio anubis*	olive baboon	XM_017948537.3	XP_017804026.1
101037414	BDNF	brain-derived neurotrophic factor	*Saimiri boliviensis*	Bolivian squirrel monkey	XM_003919940.3	XP_003919989.2
101117275	BDNF	brain-derived neurotrophic factor	*Ovis aries*	sheep	XM_012096129.4	XP_011951519.2
101134399	BDNF	brain-derived neurotrophic factor	*Gorilla gorilla*	western gorilla	XM_004050851.2	XP_004050899.1
101281702	BDNF	brain-derived neurotrophic factor	*Orcinus orca*	killer whale	XM_004263941.3	XP_004263989.1
101338565	BDNF	brain-derived neurotrophic factor	*Tursiops truncatus*	common bottlenose dolphin	XM_019947883.2	XP_019803442.1
101350169	LOC101350169	brain-derived neurotrophic factor	*Trichechus manatus latirostris*	Florida manatee	XM_004369733.1	XP_004369790.1
101372507	BDNF	brain-derived neurotrophic factor	*Odobenus rosmarus divergens*	Pacific walrus	XM_004408653.1	XP_004408710.1
101398458	LOC101398458	brain-derived neurotrophic factor	*Ceratotherium simum simum*	southern white rhinoceros	XM_004418542.2	XP_004418599.1
101428552	BDNF	brain-derived neurotrophic factor	*Dasypus novemcinctus*	nine-banded armadillo	XM_012523604.2	XP_012379058.1
101528222	BDNF	brain-derived neurotrophic factor	*Ochotona princeps*	American pika	XM_004585440.1	XP_004585497.1
101553155	BDNF	brain-derived neurotrophic factor	*Sorex araneus*	European shrew	XM_004607860.1	XP_004607917.1
101566518	Bdnf	brain-derived neurotrophic factor	*Octodon degus*	degu	XM_004644113.2	XP_004644170.1
101600646	Bdnf	brain-derived neurotrophic factor	*Jaculus jaculus*	lesser Egyptian jerboa	XM_004660846.2	XP_004660903.1
101632951	BDNF	brain-derived neurotrophic factor	*Condylura cristata*	star-nosed mole	XM_004682964.2	XP_004683021.1
101643198	BDNF	brain-derived neurotrophic factor	*Echinops telfairi*	small Madagascar hedgehog	XM_004708253.2	XP_004708310.1
101687038	BDNF	brain-derived neurotrophic factor	*Mustela putorius furo*	domestic ferret	XM_004755891.2	XP_004755948.1
101704465	Bdnf	brain-derived neurotrophic factor	*Heterocephalus glaber*	naked mole-rat	XM_004851581.3	XP_004851638.1
101837384	Bdnf	brain-derived neurotrophic factor	*Mesocricetus auratus*	golden hamster	XM_005064810.4	XP_005064867.1
101954451	Bdnf	brain-derived neurotrophic factor	*Ictidomys tridecemlineatus*	thirteen-lined ground squirrel	XM_040284582.1	XP_040140516.1
101993181	Bdnf	brain-derived neurotrophic factor	*Microtus ochrogaster*	prairie vole	XM_005364052.2	XP_005364109.1
102016703	Bdnf	brain-derived neurotrophic factor	*Chinchilla lanigera*	long-tailed chinchilla	XM_005401751.2	XP_005401808.1
102126294	BDNF	brain-derived neurotrophic factor	*Macaca fascicularis*	crab-eating macaque	XM_005578346.2	XP_005578403.1
102180782	BDNF	brain-derived neurotrophic factor	*Capra hircus*	goat	XM_005690025.2	XP_005690082.2
102247090	BDNF	brain-derived neurotrophic factor	*Myotis brandtii*	Brandt’s bat	XM_014545223.1	XP_014400709.1
102279714	BDNF	brain-derived neurotrophic factor	*Bos mutus*	wild yak	XM_005890667.2	XP_005890729.1
102395046	BDNF	brain-derived neurotrophic factor	*Bubalus bubalis*	water buffalo	XM_006068006.2	XP_006068068.1
102440705	BDNF	brain-derived neurotrophic factor	*Myotis lucifugus*	little brown bat	XM_023756374.1	XP_023612142.1
102475289	BDNF	brain-derived neurotrophic factor	*Tupaia chinensis*	Chinese tree shrew	XM_014591303.2	XP_014446789.1
102511705	BDNF	brain-derived neurotrophic factor	*Camelus ferus*	Wild Bactrian camel	XM_006195083.3	XP_006195145.1
102532564	BDNF	brain-derived neurotrophic factor	*Vicugna pacos*	alpaca	XM_031681074.1	XP_031536934.1
102730927	BDNF	brain-derived neurotrophic factor	*Leptonychotes weddellii*	Weddell seal	XM_031025064.1	XP_030880924.1
102754946	BDNF	brain-derived neurotrophic factor	*Myotis davidii*		XM_006753948.2	XP_006754011.1
102839210	BDNF	brain-derived neurotrophic factor	*Chrysochloris asiatica*	Cape golden mole	XM_006864851.1	XP_006864913.1
102870215	BDNF	brain-derived neurotrophic factor	*Elephantulus edwardii*	Cape elephant shrew	XM_006883713.1	XP_006883775.1
102896087	BDNF	brain-derived neurotrophic factor	*Pteropus alecto*	black flying fox	XM_006907988.2	XP_006908050.1
102911929	Bdnf	brain-derived neurotrophic factor	*Peromyscus maniculatus bairdii*	prairie deer mouse	XM_006980818.2	XP_006980880.1
102958723	BDNF	brain-derived neurotrophic factor	*Panthera tigris altaica*	Amur tiger	XM_015544869.1	XP_015400355.1
102977934	BDNF	brain-derived neurotrophic factor	*Physeter catodon*	sperm whale	XM_028501134.1	XP_028356935.1
103017938	BDNF	brain-derived neurotrophic factor	*Balaenoptera acutorostrata* *scammoni*		XM_007180991.1	XP_007181053.1
103085069	BDNF	brain-derived neurotrophic factor	*Lipotes vexillifer*	Yangtze River dolphin	XM_007461679.1	XP_007461741.1
103126099	BDNF	brain-derived neurotrophic factor	*Erinaceus europaeus*	western European hedgehog	XM_016194241.1	XP_016049727.1
103206496	BDNF	brain-derived neurotrophic factor	*Orycteropus afer afer*		XM_007951952.1	XP_007950143.1
103238537	BDNF	brain-derived neurotrophic factor	*Chlorocebus sabaeus*	green monkey	XM_008003703.2	XP_008001894.1
103252117	BDNF	brain-derived neurotrophic factor	*Carlito syrichta*	Philippine tarsier	XM_008050727.1	XP_008048918.1
103292699	BDNF	brain-derived neurotrophic factor	*Eptesicus fuscus*	big brown bat	XM_008148947.2	XP_008147169.1
103552694	BDNF	brain-derived neurotrophic factor	*Equus przewalskii*	Przewalski’s horse	XM_008522855.1	XP_008521077.1
103599960	BDNF	brain-derived neurotrophic factor	*Galeopterus variegatus*	Sunda flying lemur	XM_008584165.1	XP_008582387.1
103659367	BDNF	brain-derived neurotrophic factor	*Ursus maritimus*	polar bear	XM_008686880.2	XP_008685102.2
103748489	Bdnf	brain-derived neurotrophic factor	*Nannospalax galili*	Upper Galilee mountains blind mole-rat	XM_029555090.1	XP_029410950.1
104671126	BDNF	brain-derived neurotrophic factor	*Rhinopithecus roxellana*	golden snub-nosed monkey	XM_010374529.2	XP_010372831.1
104873642	Bdnf	brain-derived neurotrophic factor	*Fukomys damarensis*	Damara mole-rat	XM_033764060.1	XP_033619951.1
104982427	BDNF	brain-derived neurotrophic factor	*Bison bison bison*		XM_010831788.1	XP_010830090.1
105076551	BDNF	brain-derived neurotrophic factor	*Camelus bactrianus*	Bactrian camel	XM_010964743.1	XP_010963045.1
105106448	BDNF	brain-derived neurotrophic factor	*Camelus dromedarius*	Arabian camel	XM_031459858.1	XP_031315718.1
105293721	BDNF	brain-derived neurotrophic factor	*Pteropus vampyrus*	large flying fox	XM_011362565.2	XP_011360867.1
105463281	BDNF	brain-derived neurotrophic factor	*Macaca nemestrina*	pig-tailed macaque	XM_011710310.2	XP_011708612.1
105507710	BDNF	brain-derived neurotrophic factor	*Colobus angolensis* palliatus		XM_011936307.1	XP_011791697.1
105528120	BDNF	brain-derived neurotrophic factor	*Mandrillus leucophaeus*	drill	XM_011964715.1	XP_011820105.1
105572781	BDNF	brain-derived neurotrophic factor	*Cercocebus atys*	sooty mangabey	XM_012031697.1	XP_011887087.1
105717219	BDNF	brain-derived neurotrophic factor	*Aotus* nancymaae	Ma’s night monkey	XM_012452238.1	XP_012307661.1
105814556	BDNF	brain-derived neurotrophic factor	*Propithecus coquereli*	Coquerel’s sifaka	XM_012649556.1	XP_012505010.1
105860148	BDNF	brain-derived neurotrophic factor	*Microcebus murinus*	gray mouse lemur	XM_020286534.1	XP_020142123.1
105988089	Bdnf	brain-derived neurotrophic factor	*Dipodomys ordii*	Ord’s kangaroo rat	XM_013019592.1	XP_012875046.1
106824927	BDNF	brain-derived neurotrophic factor	*Equus asinus*	ass	XM_014831462.1	XP_014686948.1
106970278	BDNF	brain-derived neurotrophic factor	*Acinonyx jubatus*	cheetah	XM_027072960.1	XP_026928761.1
107134338	Bdnf	brain-derived neurotrophic factor	*Marmota marmota marmota*	Alpine marmot	XM_015476449.1	XP_015331935.1
107512170	BDNF	brain-derived neurotrophic factor	*Rousettus aegyptiacus*	Egyptian rousette	XM_036221046.1	XP_036076939.1
107531942	BDNF	brain-derived neurotrophic factor	*Miniopterus natalensis*		XM_016206154.1	XP_016061640.1
108311306	BDNF	brain-derived neurotrophic factor	*Cebus imitator*	Panamanian white-faced capuchin	XM_017538722.1	XP_017394211.1
108401589	BDNF	brain-derived neurotrophic factor	*Manis javanica*	Malayan pangolin	XM_017667612.2	XP_017523101.2
108519651	BDNF	brain-derived neurotrophic factor	*Rhinopithecus bieti*	black snub-nosed monkey	XM_017858805.1	XP_017714294.1
109271282	BDNF	brain-derived neurotrophic factor	*Panthera pardus*	leopard	XM_019456577.1	XP_019312122.1
109387036	BDNF	brain-derived neurotrophic factor	*Hipposideros armiger*	great roundleaf bat	XM_019650718.1	XP_019506263.1
109569212	BDNF	brain-derived neurotrophic factor	*Bos indicus*	zebu cattle	XM_019974522.1	XP_019830081.1
109695020	Bdnf	brain-derived neurotrophic factor	*Castor canadensis*	American beaver	XM_020177315.1	XP_020032904.1
110143608	BDNF	brain-derived neurotrophic factor	*Odocoileus virginianus texanus*		XM_020903256.1	XP_020758915.1
110202027	BDNF	brain-derived neurotrophic factor	*Phascolarctos cinereus*	koala	XM_020977976.1	XP_020833635.1
110285237	Bdnf	brain-derived neurotrophic factor	*Mus caroli*	Ryukyu mouse	XM_021151236.2	XP_021006895.1
110318089	Bdnf	brain-derived neurotrophic factor	*Mus pahari*	shrew mouse	XM_021192829.2	XP_021048488.1
110576669	BDNF	brain-derived neurotrophic factor	*Neomonachus schauinslandi*	Hawaiian monk seal	XM_021685880.1	XP_021541555.1
111150176	LOC111150176	brain-derived neurotrophic factor	*Enhydra lutris kenyoni*		XM_022507574.1	XP_022363282.1
111179820	BDNF	brain-derived neurotrophic factor	*Delphinapterus leucas*	beluga whale	XM_022583733.1	XP_022439441.1
111554690	BDNF	brain-derived neurotrophic factor	*Piliocolobus tephrosceles*	Ugandan red Colobus	XM_023230347.2	XP_023086115.1
112303343	BDNF	brain-derived neurotrophic factor	*Desmodus rotundus*	common vampire bat	XM_024558903.1	XP_024414671.1
112412908	BDNF	brain-derived neurotrophic factor	*Neophocaena asiaeorientalis asiaeorientalis*	Yangtze finless porpoise	XM_024764694.1	XP_024620462.1
112607193	BDNF	brain-derived neurotrophic factor	*Theropithecus gelada*	gelada	XM_025358305.1	XP_025214090.1
112667789	BDNF	brain-derived neurotrophic factor	*Canis lupus dingo*	dingo	XM_025459816.2	XP_025315601.1
112829628	BDNF	brain-derived neurotrophic factor	*Callorhinus ursinus*	northern fur seal	XM_025879326.1	XP_025735111.1
112865997	BDNF	brain-derived neurotrophic factor	*Puma concolor*	puma	XM_025929035.1	XP_025784820.1
112935019	BDNF	brain-derived neurotrophic factor	*Vulpes vulpes*	red fox	XM_026018600.1	XP_025874385.1
113199355	Bdnf	brain-derived neurotrophic factor	*Urocitellus parryii*	Arctic ground squirrel	XM_026412272.1	XP_026268057.1
113265088	BDNF	brain-derived neurotrophic factor	*Ursus arctos horribilis*		XM_026512203.1	XP_026367988.1
113624889	BDNF	brain-derived neurotrophic factor	*Lagenorhynchus obliquidens*	Pacific white-sided dolphin	XM_027115138.1	XP_026970939.1
113905455	BDNF	brain-derived neurotrophic factor	*Bos indicus x Bos taurus*	hybrid cattle	XM_027562786.1	XP_027418587.1
113913517	BDNF	brain-derived neurotrophic factor	*Zalophus californianus*	California sea lion	XM_027577807.1	XP_027433608.1
114036167	BDNF	brain-derived neurotrophic factor	*Vombatus ursinus*	common wombat	XM_027852578.1	XP_027708379.1
114093712	Bdnf	brain-derived neurotrophic factor	*Marmota flaviventris*	yellow-bellied marmot	XM_027936596.1	XP_027792397.1
114220007	BDNF	brain-derived neurotrophic factor	*Eumetopias jubatus*	Steller sea lion	XM_028117708.1	XP_027973509.1
114500881	BDNF	brain-derived neurotrophic factor	*Phyllostomus discolor*	pale spear-nosed bat	XM_028517638.2	XP_028373439.1
114620789	Bdnf	brain-derived neurotrophic factor	*Grammomys surdaster*		XM_028766921.1	XP_028622754.1
114702098	Bdnf	brain-derived neurotrophic factor	*Peromyscus leucopus*	white-footed mouse	XM_037204438.1	XP_037060333.1
114886864	BDNF	brain-derived neurotrophic factor	*Monodon monoceros*	narwhal	XM_029207934.1	XP_029063767.1
115306639	BDNF	brain-derived neurotrophic factor	*Suricata suricatta*	meerkat	XM_029957152.1	XP_029813012.1
115525821	BDNF	brain-derived neurotrophic factor	*Lynx canadensis*	Canada lynx	XM_030333208.1	XP_030189068.1
115857612	BDNF	brain-derived neurotrophic factor	*Globicephala melas*	long-finned pilot whale	XM_030864833.1	XP_030720693.1
116090620	Bdnf	brain-derived neurotrophic factor	*Mastomys coucha*	southern multimammate mouse	XM_031371313.1	XP_031227173.1
116476405	BDNF	brain-derived neurotrophic factor	*Hylobates moloch*	silvery gibbon	XM_032166370.1	XP_032022261.1
116555191	BDNF	brain-derived neurotrophic factor	*Sapajus apella*	tufted capuchin	XM_032283691.1	XP_032139582.1
116599082	BDNF	brain-derived neurotrophic factor	*Mustela erminea*	ermine	XM_032358760.1	XP_032214651.1
116638304	BDNF	brain-derived neurotrophic factor	*Phoca vitulina*	harbor seal	XM_032414183.1	XP_032270074.1
116758559	BDNF	brain-derived neurotrophic factor	*Phocoena sinus*	vaquita	XM_032641481.1	XP_032497372.1
116881443	BDNF	brain-derived neurotrophic factor	*Lontra canadensis*	Northern American river otter	XM_032881246.1	XP_032737137.1
116901726	Bdnf	brain-derived neurotrophic factor	*Rattus rattus*	black rat	XM_032903861.1	XP_032759752.1
117029758	BDNF	brain-derived neurotrophic factor	*Rhinolophus ferrumequinum*	greater horseshoe bat	XM_033118944.1	XP_032974835.1
117080419	BDNF	brain-derived neurotrophic factor	*Trachypithecus francoisi*	Francois’s langur	XM_033205475.1	XP_033061366.1
117704234	Bdnf	brain-derived neurotrophic factor	*Arvicanthis niloticus*	African grass rat	XM_034496309.1	XP_034352200.1
118015716	BDNF	brain-derived neurotrophic factor	*Mirounga leonina*	Southern elephant seal	XM_035012477.1	XP_034868368.1
118546649	BDNF	brain-derived neurotrophic factor	*Halichoerus grypus*	gray seal	XM_036109493.1	XP_035965386.1
118582264	Bdnf	brain-derived neurotrophic factor	*Onychomys torridus*	southern grasshopper mouse	XM_036184983.1	XP_036040876.1
118628031	BDNF	brain-derived neurotrophic factor	*Molossus molossus*	Pallas’s mastiff bat	XM_036258707.1	XP_036114600.1
118664943	BDNF	brain-derived neurotrophic factor	*Myotis myotis*		XM_036325692.1	XP_036181585.1
118713266	BDNF	brain-derived neurotrophic factor	*Pipistrellus kuhlii*	Kuhl’s pipistrelle	XM_036427676.1	XP_036283569.1
118853508	BDNF	brain-derived neurotrophic factor	*Trichosurus vulpecula*	common brushtail	XM_036763631.1	XP_036619526.1
118900201	BDNF	brain-derived neurotrophic factor	*Balaenoptera musculus*	Blue whale	XM_036862552.1	XP_036718447.1
118909009	BDNF	brain-derived neurotrophic factor	*Manis pentadactyla*	Chinese pangolin	XM_036879079.1	XP_036734974.1
118983123	BDNF	brain-derived neurotrophic factor	*Sturnira hondurensis*		XM_037040421.1	XP_036896316.1
119049902	BDNF	brain-derived neurotrophic factor	*Artibeus jamaicensis*	Jamaican fruit-eating bat	XM_037146035.1	XP_037001930.1
119255407	BDNF	brain-derived neurotrophic factor	*Talpa occidentalis*	Iberian mole	XM_037522538.1	XP_037378435.1
119536361	BDNF	brain-derived neurotrophic factor	*Choloepus didactylus*	southern two-toed sloth	XM_037839114.1	XP_037695042.1
119815449	Bdnf	brain-derived neurotrophic factor	*Arvicola amphibius*	Eurasian water vole	XM_038331579.1	XP_038187507.1
119943514	BDNF	brain-derived neurotrophic factor	*Tachyglossus aculeatus*	Australian echidna	XM_038764545.1	XP_038620473.1
120220096	BDNF	brain-derived neurotrophic factor	*Hyaena hyaena*	striped hyena	XM_039217098.1	XP_039073029.1
120605221	BDNF	brain-derived neurotrophic factor	*Pteropus giganteus*	Indian flying fox	XM_039866065.1	XP_039721999.1
120876831	BDNF	brain-derived neurotrophic factor	*Oryx dammah*	scimitar-horned oryx	XM_040258919.1	XP_040114853.1
121043625	BDNF	brain-derived neurotrophic factor	*Puma yagouaroundi*	jaguarundi	XM_040495572.1	XP_040351506.1
121156520	BDNF	brain-derived neurotrophic factor	*Ochotona curzoniae*	black-lipped pika	XM_040980297.1	XP_040836231.1
121465693	Bdnf	brain-derived neurotrophic factor	*Microtus oregoni*	creeping vole	XM_041679035.1	XP_041534969.1
121476611	BDNF	brain-derived neurotrophic factor	*Vulpes lagopus*	Arctic fox	XM_041730708.1	XP_041586642.1

### Data cleaning

We used the protein sequence and full gene transcripts to derive coding sequences (CDS) (via a custom script, scripts/codons.py). However, this process was met with errors in 20 “PREDICTED” protein sequences, which had invalid characters such as sequences, which have incorrect “X”, or unresolved amino acids and these sequences were subsequently exempt from the analysis. This process removes low-quality protein sequences from analysis which may inflate rates of nonsynonymous change.

### Analysis of orthologous collections (AOC): alignment, recombination detection, tree inference, and selection algorithms

The analysis of orthologous collections (AOC) application is designed for comprehensive protein-coding molecular sequence analysis (https://github.com/aglucaci/AnalysisOfOrthologousCollections). It accomplishes this through a series of comparative evolutionary methods. AOC allows for the inclusion of recombination detection, a powerful force in shaping gene evolution and interpreting analytic results. As well, it allows for lineage assignment and annotation. This feature (lineage assignment) allows between-group comparisons of selective pressures. This application currently accepts two input files: a protein sequence unaligned fasta file, and a transcript sequence unaligned fasta file for the same gene. Typically, this can be retrieved from public databases such as NCBI Orthologs. Although other methods of data compilation are also acceptable. In addition, the application is easily modifiable to accept a single CDS input, if that data is available.

If protein and transcript files are provided, a custom script “scripts/codons.py” is executed and returns coding sequences where available. Note that this script currently is set to use the standard genetic code, this will need to be modified for alternate codon tables. This script also removes “low-quality” sequences if no match is found, see the above Data cleaning section.

**Step 1. Alignment**. We used the HyPhy [46] codon-aware multiple sequence alignment procedure available at (https://github.com/veg/hyphy-analyses/tree/master/codon-msa). This was performed with a Human BDNF coding sequence *NM_001709.5 Homo sapiens brain-derived neurotrophic factor (BDNF), transcript variant 4, mRNA* as a reference-based alignment. Our alignment procedure retained 126 unique in-frame sequences.

**Step 2. Recombination detection**. Performed manually via RDP v5 [47], see below, the “Recombination detection” section for additional details. A recombination-free file is placed in the following folder: results/BDNF/Recombinants. For the purpose of this study, we did not detect recombination in our dataset.

**Step 3. Tree inference and selection analyses**. For the recombination-free fasta file, we perform maximum-likelihood phylogenetic inference via IQ-TREE [48]. Next, the recombination-free alignment and an unrooted phylogenetic tree is evaluated through a standard suite of molecular evolutionary methods. This set of selection analyses includes the following but for the sake of brevity, some of these results were not shown (essentially, most were not statistically significant or not meaningful as relevant to the evolutionary results presented here).

- FEL: locates codon sites with evidence of pervasive positive diversifying or negative selection [44].
- BUSTEDS: tests for gene-wide episodic selection [49].
- MEME: locates codon sites with evidence of episodic positive diversifying selection [50].
- aBSREL: tests if a positive selection has occurred on a proportion of branches [51].
- SLAC: performs substitution mapping [44].
- BGM: identifies groups of sites that are apparently coevolving [45].
- RELAX: compare gene-wide selection pressure between the query clade and background sequences [52].
- CFEL: comparison site-by-site selection pressure between query and background sequences [53].
- FMM: examines model fit by permitting multiple instantaneous substitutions [54].

**Step 4A**. Lineage assignment and tree annotation. For the unrooted phylogenetic tree, we perform lineage discovery, via NCBI and the python package ete3 toolkit. Assigning lineages to a K (by default, K = 20) number of taxonomic groups. Here, the aim is to have a broad representation of taxonomic groups, rather than the species being heavily clustered into a single group. As a reasonable approximation, we aim for <40% of species to be assigned to any one particular taxonomic group.

**Step 4B**. We perform tree labeling via the hyphy-analyses/Label-Trees (REF, link) method. Resulting in one annotated tree per lineage designation. For the purpose of this study, we will only consider the following five lineages for additional analyses (Artiodactyla, Carnivora, Chiroptera, Glires, Primates) as they are the most populated lineages.

**Step 5**. Selection analyses on lineages. Here, the recombination-free fasta file and the set of annotated phylogenetic trees (where labeling was performed in Step 4) is provided for analysis with the RELAX and Contrast-FEL methods.

### Recombination detection

Manually tested via RDP v5.5 with modified settings as follows:

- We also included the following algorithms/analyses: RDP [55], GENECONV [56], Chimaera [57], MaxChi [58], BootScan [59] (Primary and Secondary Scan), SiScan [60] (Primary and Secondary Scan), 3Seq [61].
- Recombination events are “accepted” in cases where three or more methods are in agreement.
- We slightly modified default parameters, such that
  - Require topological evidence.
  - Polish breakpoints.
  - Check alignment consistency.
  - Sequences are linear.
  - List events detected by >2 methods.

- We manually recheck all of the events via “Recheck all identified events with all methods”.
- We manually accept events detected by >2 methods.
- The resulting alignment was saved as a distributed alignment (with recombinant regions separated).

Recombination was not detected within our Human reference-based alignment. Therefore we used the single recombination-free alignment for analyses.

## Supplementary information

Supplementary Material

## Data Availability

The AOC application is freely available via a dedicated GitHub repository at: https://github.com/aglucaci/AnalysisOfOrthologousCollections Raw data for this study is available on GitHub: https://github.com/aglucaci/AnalysisOfOrthologousCollections/tree/main/data/BDNF. Full results for this study include all HyPhy selection analyses JSON-formatted result files are available on GitHub: https://github.com/aglucaci/AnalysisOfOrthologousCollections/tree/main/results/BDNF.
