# Supplementary material for: The evolution of BDNF is defined by strict purifying selection and prodomain spatial coevolution, but what does it mean for human brain disease?
Source: Transl Psychiatry. 2022 Jun 22;12:258. doi: 10.1038/s41398-022-02021-w (PMC9217794; doi:10.1038/s41398-022-02021-w)
Supplement: Supplementary file 1 — Supplementary Material [file 41398_2022_2021_MOESM1_ESM.pdf]

Supplementary Material for "The evolution of BDNF is defined by strict purifying selection and prodomain spatial coevolution, but what does it mean for human brain disease?"

Alexander G. Lucaci[1,*], Michael J. Notaras[2,*] , Sergei L. Kosakovsky Pond[1], Dilek Colak[2,3]

[1] Institute for Genomics and Evolutionary Medicine, Science & Education Research Center, Temple University, Philadelphia, Pennsylvania, USA.
[2] Center for Neurogenetics, Brain & Mind Research Institute, Weill Medical College, Cornell University, New York, New York, USA.
[3] Gale and Ira Drukier Institute for Children's Health, Weill Cornell Medical College, Cornell University, New York, NY, USA.

*Alexander G. Lucaci & Michael J. Notaras contributed equally and share first authorship.

Table S1. The FEL analysis of the BDNF gene found 174 of 261 (66.7%) sites to be statistically significant (LRT p-value <= 0.1) for pervasive negative (purifying) selection.

Site	alpha	beta	alpha=beta	LRT	p-value	Total branch length	dN/dS LB	dN/dS MLE	dN/dS UB
1	0.0000	0.0000	0.0000	0.0000	1.0000	0.0000	0.0000	0.0000	0.0000
2	0.0000	0.0000	0.0000	0.0000	1.0000	0.0000	0.0000	0.0000	0.0000
3	0.0000	0.0000	0.0000	0.0000	1.0000	0.0000	0.0000	0.0000	0.0000
4	0.1116	0.0635	0.0811	0.1573	0.6916	0.6594	0.0325	0.5690	2.5205
5	0.0000	0.0564	0.0468	0.3606	0.5482	0.3806	1462.7972	10000.0000	10000.0000
6	0.2221	0.0000	0.0805	4.0430	0.0444	0.6545	0.0000	0.0000	0.5478
7	0.0000	0.0000	0.0000	0.0000	1.0000	0.0000	0.0000	0.0000	0.0000

8	0.0000	0.0000	0.0000	0.0000	1.0000	0.0000	0.0000	0.0000	0.0000
9	0.0928	0.0000	0.0397	2.0198	0.1553	0.3227	0.0000	0.0000	1.2848
10	0.1594	0.0000	0.0409	2.7359	0.0981	0.3326	0.0000	0.0000	0.6580
11	0.1729	0.0000	0.0900	3.1185	0.0774	0.7317	0.0000	0.0000	0.9176
12	0.0000	0.0000	0.0000	0.0000	1.0000	0.0000	0.0000	0.0000	0.0000
13	1.9630	0.0000	0.2850	22.6368	0.0000	2.3174	0.0000	0.0000	0.0549
14	0.0000	0.2172	0.1438	3.0861	0.0790	1.1694	6185.5906	10000.0000	10000.0000
15	0.0000	0.0000	0.0000	0.0000	1.0000	0.0000	0.0000	0.0000	0.0000
16	0.0000	0.0000	0.0000	0.0000	1.0000	0.0000	0.0000	0.0000	0.0000
17	0.2673	0.0530	0.0885	1.1690	0.2796	0.7199	0.0113	0.1982	0.8804
18	0.3413	0.0000	0.1102	6.7673	0.0093	0.8958	0.0000	0.0000	0.3054
19	0.4344	0.0000	0.1182	7.7643	0.0053	0.9612	0.0000	0.0000	0.2399
20	0.7437	0.0000	0.1993	12.7922	0.0003	1.6204	0.0000	0.0000	0.1425
21	0.0000	0.0000	0.0000	0.0000	1.0000	0.0000	0.0000	0.0000	0.0000
22	0.7734	0.0000	0.1935	13.6496	0.0002	1.5734	0.0000	0.0000	0.1283
23	0.1435	0.0527	0.0773	0.4750	0.4907	0.6284	0.0210	0.3670	1.6325
24	1.7963	0.4672	0.8629	8.8413	0.0029	7.0170	0.1183	0.2601	0.4893

25	1.4891	0.3979	0.5537	4.2623	0.0390	4.5023	0.1213	0.2672	0.5042
26	0.1396	1.0896	0.7817	6.8872	0.0087	6.3560	4.5038	7.8049	12.5990
27	0.2849	0.2004	0.2436	0.1449	0.7035	1.9806	0.1198	0.7033	2.1145
28	0.0000	0.0000	0.0000	0.0000	1.0000	0.0000	0.0000	0.0000	0.0000
29	0.4139	0.0566	0.1834	4.1399	0.0419	1.4914	0.0078	0.1367	0.6064
30	0.0000	0.3797	0.2579	4.6268	0.0315	2.0973	7260.0520	10000.0000	10000.0000
31	0.2774	0.0000	0.0763	5.1487	0.0233	0.6206	0.0000	0.0000	0.3653
32	0.2572	0.4242	0.3976	0.2465	0.6196	3.2327	0.7845	1.6493	3.0082
33	0.3030	0.2456	0.2654	0.0484	0.8259	2.1581	0.2009	0.8105	2.1234
34	1.0536	0.1089	0.3602	9.9540	0.0016	2.9291	0.0172	0.1034	0.3222
35	0.0000	0.0000	0.0000	0.0000	1.0000	0.0000	0.0000	0.0000	0.0000
36	0.2906	0.0686	0.1107	0.9484	0.3301	0.9000	0.0134	0.2360	1.0454
37	0.8023	0.0000	0.2613	15.6626	0.0001	2.1248	0.0000	0.0000	0.1321
38	0.7113	0.3303	0.4528	1.6696	0.1963	3.6817	0.1837	0.4644	0.9547
39	0.4421	0.5250	0.5030	0.0485	0.8256	4.0903	0.5420	1.1877	2.2205
40	0.4097	0.0000	0.1578	7.5858	0.0059	1.2834	0.0000	0.0000	0.3013
41	0.0000	0.0551	0.0393	0.6774	0.4105	0.3195	1462.7591	10000.0000	10000.0000

42	1.2391	0.0506	0.2862	13.8667	0.0002	2.3274	0.0023	0.0409	0.1816
43	0.1559	0.0000	0.0396	2.7457	0.0975	0.3218	0.0000	0.0000	0.6535
44	0.3404	0.0556	0.1494	2.9408	0.0864	1.2148	0.0093	0.1634	0.7237
45	0.0928	0.0000	0.0443	1.4933	0.2217	0.3605	0.0000	0.0000	1.7490
46	0.0000	0.1640	0.1370	1.0523	0.3050	1.1140	5270.0970	10000.0000	10000.0000
47	1.8271	0.1373	0.3286	11.8421	0.0006	2.6721	0.0187	0.0751	0.1965
48	0.7118	1.0257	0.9372	0.4209	0.5165	7.6211	0.8165	1.4411	2.3527
49	1.3089	0.3744	0.5850	4.9417	0.0262	4.7570	0.1222	0.2860	0.5605
50	0.2882	0.0532	0.1161	2.0383	0.1534	0.9440	0.0105	0.1844	0.8138
51	0.1419	0.0000	0.0395	2.5243	0.1121	0.3214	0.0000	0.0000	0.7470
52	0.2736	0.3297	0.3204	0.0312	0.8599	2.6057	0.4762	1.2050	2.4754
53	1.5426	0.4637	0.8202	7.0701	0.0078	6.6692	0.1365	0.3006	0.5668
54	1.5617	0.2216	0.6252	13.3081	0.0003	5.0838	0.0440	0.1419	0.3335
55	3.6302	0.5463	1.6240	22.5001	0.0000	13.2053	0.0643	0.1505	0.2957
56	1.5021	0.0545	0.4719	20.6584	0.0000	3.8374	0.0021	0.0363	0.1612
57	0.5526	0.2782	0.3576	0.9810	0.3220	2.9080	0.1797	0.5034	1.0950
58	0.3979	0.0869	0.2313	2.3382	0.1262	1.8808	0.0124	0.2184	0.9655

59	11.1190	0.8319	1.4313	1.5693	0.2103	11.6383	0.0038	0.0748	0.6522
60	4.3720	0.3686	1.3037	33.3567	0.0000	10.6014	0.0332	0.0843	0.1727
61	0.0000	0.0000	0.0000	0.0000	1.0000	0.0000	0.0000	0.0000	0.0000
62	1.7721	2193.2857	2856.9387	0.0000	1.0000	23231.3076	0.3292	1237.7020	10000.0000
63	0.0000	3.6440	2.0642	0.9793	0.3224	16.7849	1464.9793	10000.0000	10000.0000
64	7.4868	0.0000	1.0937	6.3427	0.0118	8.8931	0.0000	0.0000	0.1725
65	122.5088	0.0000	1.1740	7.2662	0.0070	9.5464	0.0000	0.0000	0.0108
66	2.8095	0.0000	0.7275	4.7774	0.0288	5.9161	0.0000	0.0000	0.3619
67	10.8121	0.0000	1.6286	13.5042	0.0002	13.2432	0.0000	0.0000	0.0777
68	4.1586	0.4872	1.5564	8.0811	0.0045	12.6556	0.0193	0.1172	0.3731
69	3.2764	0.3962	1.3489	22.0059	0.0000	10.9685	0.0431	0.1209	0.2630
70	0.9289	0.0000	0.3524	13.2397	0.0003	2.8657	0.0000	0.0000	0.1693
71	1.1053	0.0000	0.3383	20.8817	0.0000	2.7513	0.0000	0.0000	0.0943
72	0.2921	0.0985	0.1261	0.6630	0.4155	1.0251	0.0558	0.3373	1.0461
73	1.1053	0.0000	0.3395	20.5444	0.0000	2.7603	0.0000	0.0000	0.0954
74	2.9165	0.0575	0.6252	32.3768	0.0000	5.0841	0.0011	0.0197	0.0877
75	0.8228	0.0000	0.2007	13.9151	0.0002	1.6320	0.0000	0.0000	0.1241

76	5.5185	0.0000	0.5700	53.7705	0.0000	4.6353	0.0000	0.0000	0.0174
77	0.6280	0.0619	0.2215	5.6574	0.0174	1.8010	0.0056	0.0985	0.4366
78	2.1641	0.0489	0.3787	23.0125	0.0000	3.0792	0.0013	0.0226	0.1005
79	1.8388	0.0000	0.4162	29.0685	0.0000	3.3840	0.0000	0.0000	0.0556
80	0.2839	0.0541	0.0909	1.2074	0.2718	0.7393	0.0109	0.1905	0.8475
81	1.3529	0.0000	0.5463	20.2275	0.0000	4.4420	0.0000	0.0000	0.1126
82	1.8857	0.0830	0.7606	19.4503	0.0000	6.1851	0.0025	0.0440	0.1950
83	2.4273	0.1547	0.5523	20.6750	0.0000	4.4910	0.0157	0.0637	0.1663
84	0.5934	0.0000	0.0911	7.3673	0.0066	0.7411	0.0000	0.0000	0.1756
85	0.3406	0.1489	0.1729	0.4375	0.5083	1.4059	0.1081	0.4371	1.1399
86	0.0000	0.3235	0.2567	2.3672	0.1239	2.0875	6809.7393	10000.0000	10000.0000
87	1.2767	0.1562	0.4047	9.9233	0.0016	3.2907	0.0303	0.1223	0.3201
88	1.3410	0.5707	0.8242	3.2658	0.0707	6.7019	0.2024	0.4256	0.7810
89	1.2511	0.1289	0.5092	12.0135	0.0005	4.1408	0.0170	0.1031	0.3194
90	0.6067	0.0552	0.2020	6.0849	0.0136	1.6429	0.0052	0.0910	0.4030
91	4.6374	1.7289	2.1855	6.6899	0.0097	17.7717	0.2477	0.3728	0.5401
92	1.8962	0.0535	0.4607	23.2267	0.0000	3.7463	0.0016	0.0282	0.1245

93	3.5662	0.0542	0.7407	40.9892	0.0000	6.0226	0.0009	0.0152	0.0676
94	1.3252	0.3547	0.4855	3.6636	0.0556	3.9476	0.1139	0.2676	0.5226
95	1.3323	1.5991	1.5317	0.1473	0.7011	12.4555	0.7548	1.2003	1.8159
96	1.3529	0.0000	0.1787	14.6186	0.0001	1.4535	0.0000	0.0000	0.0749
97	1.8434	0.0489	0.2953	17.5494	0.0000	2.4011	0.0015	0.0265	0.1178
98	0.6510	0.0536	0.2046	6.5994	0.0102	1.6636	0.0047	0.0824	0.3692
99	0.0000	0.0000	0.0000	0.0000	1.0000	0.0000	0.0000	0.0000	0.0000
100	0.5931	0.3419	0.4207	0.4996	0.4797	3.4205	0.1807	0.5764	1.3251
101	5.6241	0.0678	0.7121	36.3681	0.0000	5.7902	0.0007	0.0120	0.0536
102	2.4986	0.1708	0.7664	25.4012	0.0000	6.2322	0.0169	0.0684	0.1784
103	0.6960	0.0000	0.2116	11.7725	0.0006	1.7204	0.0000	0.0000	0.1687
104	2.3048	0.0000	0.5043	34.9881	0.0000	4.1010	0.0000	0.0000	0.0444
105	0.6641	0.0000	0.1786	10.1702	0.0014	1.4520	0.0000	0.0000	0.1795
106	0.0000	0.0000	0.0000	0.0000	1.0000	0.0000	0.0000	0.0000	0.0000
107	0.9618	0.0000	0.3094	15.6026	0.0001	2.5158	0.0000	0.0000	0.1311
108	1.7507	0.0000	0.2683	25.8194	0.0000	2.1813	0.0000	0.0000	0.0495
109	1.2561	0.0472	0.2636	13.7621	0.0002	2.1434	0.0021	0.0376	0.1661

110	0.3333	0.0000	0.0900	5.1186	0.0237	0.7317	0.0000	0.0000	0.3586
111	0.0000	0.0000	0.0000	0.0000	1.0000	0.0000	0.0000	0.0000	0.0000
112	0.2221	0.0000	0.0736	4.3993	0.0360	0.5984	0.0000	0.0000	0.4774
113	0.2700	0.0000	0.1012	3.9075	0.0481	0.8232	0.0000	0.0000	0.5785
114	0.6015	0.0000	0.0925	7.5591	0.0060	0.7518	0.0000	0.0000	0.1729
115	2.1843	0.0000	0.5950	37.4897	0.0000	4.8380	0.0000	0.0000	0.0485
116	1.7115	0.0000	0.4751	29.9207	0.0000	3.8634	0.0000	0.0000	0.0619
117	1.3529	0.0000	0.4841	22.7688	0.0000	3.9361	0.0000	0.0000	0.0965
118	0.2799	0.0000	0.0871	4.6517	0.0310	0.7081	0.0000	0.0000	0.4354
119	2.6166	0.0000	0.5451	36.1605	0.0000	4.4325	0.0000	0.0000	0.0423
120	0.0928	0.0000	0.0445	1.5555	0.2123	0.3619	0.0000	0.0000	1.7079
121	3.0538	0.0000	0.9175	44.7049	0.0000	7.4605	0.0000	0.0000	0.0424
122	0.0000	0.0000	0.0000	0.0000	1.0000	0.0000	0.0000	0.0000	0.0000
123	0.5018	0.0000	0.1210	8.4758	0.0036	0.9840	0.0000	0.0000	0.2034
124	0.0000	0.0000	0.0000	0.0000	1.0000	0.0000	0.0000	0.0000	0.0000
125	0.0000	0.0000	0.0000	0.0000	1.0000	0.0000	0.0000	0.0000	0.0000
126	0.1674	0.0519	0.0785	0.6645	0.4150	0.6381	0.0174	0.3101	1.3547

127	0.5910	0.0680	0.1651	3.2197	0.0728	1.3427	0.0066	0.1151	0.5099
128	0.4829	0.0000	0.2240	7.5898	0.0059	1.8217	0.0000	0.0000	0.3344
129	0.7281	0.0000	0.1574	12.1458	0.0005	1.2801	0.0000	0.0000	0.1324
130	1.3529	0.0000	0.4223	26.4290	0.0000	3.4343	0.0000	0.0000	0.0770
131	1.3529	0.0000	0.4072	24.6703	0.0000	3.3113	0.0000	0.0000	0.0770
132	0.5994	0.0000	0.0843	7.7423	0.0054	0.6853	0.0000	0.0000	0.1579
133	0.0000	0.0000	0.0000	0.0000	1.0000	0.0000	0.0000	0.0000	0.0000
134	2.4575	0.0000	0.6712	37.7462	0.0000	5.4579	0.0000	0.0000	0.0483
135	0.0000	0.0435	0.0438	0.0008	0.9774	0.3558	1462.1672	10000.0000	10000.0000
136	1.0696	0.0000	0.2395	17.4870	0.0000	1.9473	0.0000	0.0000	0.0925
137	0.4335	0.0000	0.1301	7.1875	0.0073	1.0578	0.0000	0.0000	0.2750
138	0.6509	0.0000	0.2392	11.8685	0.0006	1.9451	0.0000	0.0000	0.1867
139	0.4217	0.0000	0.1161	7.6814	0.0056	0.9442	0.0000	0.0000	0.2443
140	0.9836	0.0000	0.1277	11.8927	0.0006	1.0384	0.0000	0.0000	0.0967
141	3.5104	0.0000	0.9082	50.5522	0.0000	7.3849	0.0000	0.0000	0.0344
142	0.0000	0.0000	0.0000	0.0000	1.0000	0.0000	0.0000	0.0000	0.0000
143	3.8890	0.0000	0.8487	57.0286	0.0000	6.9012	0.0000	0.0000	0.0273

144	0.5803	0.0000	0.1576	10.3294	0.0013	1.2818	0.0000	0.0000	0.1796
145	0.8551	0.0000	0.2336	15.3582	0.0001	1.8993	0.0000	0.0000	0.1209
146	3.1941	0.0000	0.8296	52.1403	0.0000	6.7458	0.0000	0.0000	0.0335
147	0.4576	0.0000	0.1172	8.0791	0.0045	0.9527	0.0000	0.0000	0.2215
148	0.0000	0.0000	0.0000	0.0000	1.0000	0.0000	0.0000	0.0000	0.0000
149	0.1879	0.0000	0.0883	2.9817	0.0842	0.7177	0.0000	0.0000	0.8603
150	2.4574	0.0000	0.2729	29.1158	0.0000	2.2192	0.0000	0.0000	0.0347
151	1.3529	0.0000	0.3641	20.8617	0.0000	2.9610	0.0000	0.0000	0.0881
152	1.8431	0.0000	0.3369	26.2702	0.0000	2.7398	0.0000	0.0000	0.0543
153	0.0000	0.0000	0.0000	0.0000	1.0000	0.0000	0.0000	0.0000	0.0000
154	1.1326	0.0449	0.2254	12.6183	0.0004	1.8326	0.0023	0.0397	0.1759
155	1.9612	0.1105	0.5215	19.7057	0.0000	4.2410	0.0093	0.0564	0.1754
156	9.5442	0.0000	0.6185	72.6680	0.0000	5.0296	0.0000	0.0000	0.0090
157	0.5748	0.0547	0.1375	3.7909	0.0515	1.1182	0.0054	0.0951	0.4215
158	0.0000	0.0000	0.0000	0.0000	1.0000	0.0000	0.0000	0.0000	0.0000
159	2.8095	0.0000	0.8095	44.3396	0.0000	6.5824	0.0000	0.0000	0.0424
160	2.4575	0.0000	0.6733	41.9754	0.0000	5.4754	0.0000	0.0000	0.0429

161	3.1758	0.0000	0.7134	48.2008	0.0000	5.8007	0.0000	0.0000	0.0328
162	1.3529	0.0000	0.3849	24.2341	0.0000	3.1298	0.0000	0.0000	0.0770
163	1.6026	0.0501	0.3633	19.2676	0.0000	2.9542	0.0018	0.0313	0.1388
164	0.6094	0.0000	0.1545	10.8904	0.0010	1.2565	0.0000	0.0000	0.1629
165	0.2724	0.0000	0.0441	3.6202	0.0571	0.3588	0.0000	0.0000	0.3729
166	1.3529	0.0000	0.3828	23.3448	0.0000	3.1125	0.0000	0.0000	0.0779
167	1.3492	0.0541	0.4779	20.1541	0.0000	3.8862	0.0023	0.0401	0.1781
168	0.0000	0.0000	0.0000	0.0000	1.0000	0.0000	0.0000	0.0000	0.0000
169	0.0000	0.0000	0.0000	0.0000	1.0000	0.0000	0.0000	0.0000	0.0000
170	0.0000	0.0000	0.0000	0.0000	1.0000	0.0000	0.0000	0.0000	0.0000
171	2.3512	0.0000	0.6698	30.8621	0.0000	5.4463	0.0000	0.0000	0.0603
172	1.3529	0.0000	0.3510	23.6433	0.0000	2.8544	0.0000	0.0000	0.0750
173	0.9159	0.0000	0.2362	16.1752	0.0001	1.9207	0.0000	0.0000	0.1110
174	2.5514	0.0000	0.5850	39.0254	0.0000	4.7569	0.0000	0.0000	0.0413
175	0.4238	0.0000	0.1290	7.0778	0.0078	1.0492	0.0000	0.0000	0.2813
176	3.0000	0.0000	0.8020	50.0929	0.0000	6.5213	0.0000	0.0000	0.0351
177	0.2903	0.0000	0.0867	4.8037	0.0284	0.7050	0.0000	0.0000	0.4106

178	2.0769	0.0000	0.7435	33.6840	0.0000	6.0457	0.0000	0.0000	0.0637
179	1.3529	0.0000	0.2916	20.9924	0.0000	2.3712	0.0000	0.0000	0.0756
180	2.8095	0.0000	0.5579	41.9086	0.0000	4.5370	0.0000	0.0000	0.0356
181	0.4269	0.0000	0.1294	7.1083	0.0077	1.0522	0.0000	0.0000	0.2792
182	2.0769	0.0000	0.5715	36.1609	0.0000	4.6469	0.0000	0.0000	0.0510
183	2.4177	0.0000	0.7387	39.0146	0.0000	6.0071	0.0000	0.0000	0.0493
184	4.3195	0.0699	1.0443	43.8566	0.0000	8.4918	0.0009	0.0162	0.0716
185	0.0000	0.0000	0.0000	0.0000	1.0000	0.0000	0.0000	0.0000	0.0000
186	0.1373	0.0000	0.0381	2.5665	0.1092	0.3095	0.0000	0.0000	0.7378
187	1.3529	0.0000	0.3201	18.1218	0.0000	2.6032	0.0000	0.0000	0.0888
188	0.0000	0.0000	0.0000	0.0000	1.0000	0.0000	0.0000	0.0000	0.0000
189	0.2720	0.0000	0.0441	3.6125	0.0573	0.3584	0.0000	0.0000	0.3735
190	1.8571	0.0000	0.3924	23.7914	0.0000	3.1908	0.0000	0.0000	0.0653
191	0.9044	0.0000	0.1654	9.9503	0.0016	1.3450	0.0000	0.0000	0.1449
192	0.0000	0.0000	0.0000	0.0000	1.0000	0.0000	0.0000	0.0000	0.0000
193	0.9156	0.0000	0.1660	10.0482	0.0015	1.3502	0.0000	0.0000	0.1431
194	0.2724	0.0000	0.0451	3.5786	0.0585	0.3665	0.0000	0.0000	0.3827

195	0.1373	0.0000	0.0392	2.5100	0.1131	0.3184	0.0000	0.0000	0.7677
196	0.8673	0.0531	0.1785	6.8492	0.0089	1.4516	0.0035	0.0612	0.2703
197	0.6237	0.0000	0.0904	7.7740	0.0053	0.7354	0.0000	0.0000	0.1619
198	0.7734	0.0000	0.1617	12.4902	0.0004	1.3145	0.0000	0.0000	0.1260
199	0.9136	0.0000	0.2416	15.6381	0.0001	1.9647	0.0000	0.0000	0.1160
200	0.0000	0.0439	0.0439	0.0088	0.9254	0.3566	1462.1862	10000.0000	10000.0000
201	0.8954	0.0000	0.2625	17.0957	0.0000	2.1349	0.0000	0.0000	0.1132
202	0.5743	0.0690	0.1664	3.1111	0.0778	1.3533	0.0068	0.1202	0.5326
203	3.0000	0.0000	0.8142	50.6222	0.0000	6.6207	0.0000	0.0000	0.0351
204	0.4000	0.0000	0.0825	6.2389	0.0125	0.6712	0.0000	0.0000	0.2512
205	1.2072	0.0537	0.2628	11.6100	0.0007	2.1373	0.0025	0.0445	0.1973
206	1.3529	0.0000	0.3132	21.2564	0.0000	2.5470	0.0000	0.0000	0.0750
207	0.2877	0.0000	0.0444	3.7112	0.0540	0.3610	0.0000	0.0000	0.3519
208	2.6056	0.0000	0.5748	39.9407	0.0000	4.6744	0.0000	0.0000	0.0376
209	0.5731	0.0000	0.1538	10.3900	0.0013	1.2509	0.0000	0.0000	0.1770
210	0.5502	0.0000	0.0759	7.9203	0.0049	0.6170	0.0000	0.0000	0.1531
211	0.0000	0.0000	0.0000	0.0000	1.0000	0.0000	0.0000	0.0000	0.0000

212	1.6439	0.0000	0.3321	24.7877	0.0000	2.7006	0.0000	0.0000	0.0609
213	0.3655	0.1001	0.1571	1.5333	0.2156	1.2773	0.0454	0.2740	0.8525
214	4.5596	0.0000	0.6112	53.3306	0.0000	4.9697	0.0000	0.0000	0.0213
215	0.0000	0.0000	0.0000	0.0000	1.0000	0.0000	0.0000	0.0000	0.0000
216	1.8153	0.0000	0.2159	20.6059	0.0000	1.7556	0.0000	0.0000	0.0522
217	0.9243	0.0000	0.2666	14.5720	0.0001	2.1675	0.0000	0.0000	0.1312
218	2.0769	0.0000	0.3184	21.3914	0.0000	2.5891	0.0000	0.0000	0.0590
219	0.6452	0.0000	0.0910	7.8870	0.0050	0.7397	0.0000	0.0000	0.1567
220	0.0928	0.0000	0.0395	1.1939	0.2746	0.3210	0.0000	0.0000	1.7623
221	0.0000	0.0000	0.0000	0.0000	1.0000	0.0000	0.0000	0.0000	0.0000
222	0.2835	0.0000	0.0789	5.0825	0.0242	0.6414	0.0000	0.0000	0.3719
223	2.3567	0.0000	0.3682	24.3183	0.0000	2.9942	0.0000	0.0000	0.0521
224	1.6066	0.0000	0.4605	22.0347	0.0000	3.7447	0.0000	0.0000	0.0858
225	11.2648	0.0000	1.0274	68.4271	0.0000	8.3540	0.0000	0.0000	0.0116
226	0.7404	0.0000	0.2181	12.0620	0.0005	1.7737	0.0000	0.0000	0.1610
227	0.1729	0.0000	0.0784	3.7735	0.0521	0.6379	0.0000	0.0000	0.7082
228	1.7586	0.0000	0.4540	30.8744	0.0000	3.6920	0.0000	0.0000	0.0593

229	2.3195	0.0000	0.6722	35.6279	0.0000	5.4661	0.0000	0.0000	0.0530
230	0.4314	0.0000	0.1189	7.6758	0.0056	0.9671	0.0000	0.0000	0.2444
231	0.0000	0.0000	0.0000	0.0000	1.0000	0.0000	0.0000	0.0000	0.0000
232	0.0000	0.0000	0.0000	0.0000	1.0000	0.0000	0.0000	0.0000	0.0000
233	0.5711	0.1372	0.1973	2.0310	0.1541	1.6047	0.0597	0.2403	0.6289
234	0.0000	0.0000	0.0000	0.0000	1.0000	0.0000	0.0000	0.0000	0.0000
235	0.2636	0.0000	0.0440	3.5732	0.0587	0.3576	0.0000	0.0000	0.3853
236	2.6511	0.0000	0.7753	46.0604	0.0000	6.3044	0.0000	0.0000	0.0390
237	1.0663	0.0560	0.3290	12.7008	0.0004	2.6757	0.0030	0.0525	0.2331
238	0.6357	0.0000	0.1581	11.0293	0.0009	1.2852	0.0000	0.0000	0.1594
239	0.0000	0.0000	0.0000	0.0000	1.0000	0.0000	0.0000	0.0000	0.0000
240	2.0993	0.0000	0.8145	34.8044	0.0000	6.6232	0.0000	0.0000	0.0635
241	0.6014	0.0000	0.0939	7.3236	0.0068	0.7636	0.0000	0.0000	0.1788
242	1.3529	0.0000	0.1970	20.0704	0.0000	1.6019	0.0000	0.0000	0.0637
243	0.4994	0.0000	0.1160	8.6482	0.0033	0.9429	0.0000	0.0000	0.1943
244	0.0000	0.0000	0.0000	0.0000	1.0000	0.0000	0.0000	0.0000	0.0000
245	0.0000	0.0000	0.0000	0.0000	1.0000	0.0000	0.0000	0.0000	0.0000

246	0.0928	0.0000	0.0366	2.1737	0.1404	0.2972	0.0000	0.0000	1.1363
247	0.7803	0.0000	0.2206	12.6274	0.0004	1.7937	0.0000	0.0000	0.1503
248	0.1649	0.0000	0.0395	2.8905	0.0891	0.3211	0.0000	0.0000	0.6024
249	1.0218	0.1248	0.4662	10.2373	0.0014	3.7913	0.0202	0.1222	0.3798
250	0.0000	0.0000	0.0000	0.0000	1.0000	0.0000	0.0000	0.0000	0.0000
251	0.0000	0.0000	0.0000	0.0000	1.0000	0.0000	0.0000	0.0000	0.0000
252	0.0000	0.0000	0.0000	0.0000	1.0000	0.0000	0.0000	0.0000	0.0000
253	0.1668	0.0000	0.0394	2.8836	0.0895	0.3208	0.0000	0.0000	0.5956
254	0.2793	0.1183	0.1771	0.8135	0.3671	1.4401	0.0704	0.4234	1.3076
255	1.2093	0.0823	0.5111	11.5180	0.0007	4.1560	0.0039	0.0680	0.3022
256	0.1388	0.0000	0.0395	2.5133	0.1129	0.3213	0.0000	0.0000	0.7658
257	0.0000	0.0000	0.0000	0.0000	1.0000	0.0000	0.0000	0.0000	0.0000
258	1.9630	0.0000	0.4167	30.3846	0.0000	3.3887	0.0000	0.0000	0.0513
259	0.9679	0.0000	0.2005	15.4029	0.0001	1.6302	0.0000	0.0000	0.1003
260	0.2929	0.0000	0.1083	5.9411	0.0148	0.8807	0.0000	0.0000	0.3766
261	0.1136	0.0508	0.0703	0.3166	0.5737	0.5720	0.0255	0.4468	1.9804

These results are also available at the following link:
https://github.com/aglucaci/AnalysisOfOrthologousCollections/blob/main/tables/BDNF/BDNF_FEL_CI.csv
